# Waste-Coffee-Derived Activated Carbon as Efficient Adsorbent for Water Treatment

**DOI:** 10.3390/ma15238684

**Published:** 2022-12-06

**Authors:** Hong-Ming Chen, Woon-Ming Lau, Dan Zhou

**Affiliations:** 1 Beijing Advanced Innovation Center for Materials Genome Engineering & Center for Green Innovation, School of Mathematics and Physics, University of Science and Technology Beijing, Beijing 100083, China; 2 Shunde Innovation School, University of Science and Technology Beijing, Foshan 528000, China

**Keywords:** rhodamine B, adsorption, waste coffee, activated carbon, surface properties

## Abstract

Activated carbon prepared from waste coffee was utilized as a potential low-cost adsorbent to remove Rhodamine B from aqueous solution. A series of physical characterizations verify that the obtained activated carbon possesses a layered and ordered hexagonal structure with a wrinkled and rough surface. In addition, high specific surface area, appropriate pore distribution, and desired surface functional groups are revealed, which promote the adsorption properties. Various adsorption experiments were conducted to investigate the effect on the absorption capacity (e.g., of initial dye concentration, temperature and solution pH) of the material. The results showed that the waste-coffee-derived activated carbon with a large surface area of approximately 952.7 m^2^ g^−1^ showed a maximum uptake capacity of 83.4 mg g^−1^ at the pH of 7 with the initial dye concentration of 100 mg L^−1^ under 50°C. The higher adsorption capacity can be attributed to the strong electrostatic attraction between the negatively charged functional groups in activated carbon and the positively charged functional groups in RB. The kinetic data and the corresponding kinetic parameters were simulated to evaluate the mechanism of the adsorption process, which can fit well with the highest R^2^. The adsorption results confirmed the promising potential of the as-prepared waste-coffee-derived activated carbon as a dye adsorbent.

## 1. Introduction

With the development of human society, the pursuit of a good environment and good health becomes more and more important. However, due to inefficient past development, environmental pollution can be seen everywhere on Earth, which severely harms humans. For example, various dyes are widely used in many industries, such as clothing, food, papermaking, cosmetics, biological stains and plastics [1,2,3,4,5,6]. Although these dyes have some desired benefits, they can be accompanied by many problems. Specifically, Rhodamine B (RB) is one of the most important dyes. It has been widely used as a fluorescent dye agent in the laboratory, in stained glass, in special fireworks and in other industries [7,8,9]. However, RB is also reported to be deleterious and carcinogenic for human beings and animals [10,11]. Moreover, it is usually stable in the environment and non-biodegradable [12]. Therefore, the removal of RB from waste water is very important for the protection of aquatic life, the environment and public health.

Many approaches have been reported for the removal of RB from waste water, such as photocatalytic degradation [13,14,15,16,17,18], liquid–liquid extraction through membrane technology [19], the electro-Fenton process [20], electrochemical oxidation [21], biodegradation [22] and microwave- or ultrasonic-assisted methods [7]. Among these, the photocatalytic degradation method has received wide attention. With this method, chemically complex organic pollutants might degrade into simpler, non-polluting molecules. However, these resultant products might not be good for wildlife because they still exist in waste water. The most direct and effective way is to remove the organic pollutants from waste by adsorption. This method is currently attracting substantial attention [23,24]. Activated carbon has shown extreme potential for dye adsorption due to its porous structure, remarkably large surface area, and in turn high adsorption properties [25]. However, due to the high cost, the application for removing dyes in industry is still limited. Thus, the synthesis of low-cost activated carbons is greatly desired. 

Coffee is one of the most popular drinks in the world, and hundreds of millions of bags of coffee are consumed every year [26]. Most of the waste coffee is treated as garbage for disposal. It will undoubtedly be an economical matter if coffee waste is used as raw material to produce activated carbon. Therefore, the exploration of an effective design and synthesis route from waste coffee to activated carbon seems to be significantly valuable. Herein, we report a simple method to prepare activated carbon with waste coffee as the starting material, aiming to propose a simple and low-cost method to produce activated carbon and simultaneously solve the recovery and utilization of waste coffee. The porous structure of the as-prepared activated carbon is characterized in detail, and then its application in removing RB from aqueous solution is investigated. This paper aims to combine the recycling of waste coffee with sewage treatment to reduce environmental pollution.

## 2. Materials and Methods

### 2.1. Activated Carbon Synthesis

Waste-coffee-derived activated carbon was prepared by a simple method. The first step involved carbonization, in which the waste coffee (4.0 g) was dried at 80°C for 10 h and then annealed at 900 °C for 1 h in argon with a heating rate of 5 °C min^−1^. The second step was pore forming, in which the above as-prepared sample (2.0 g) and KOH (2.0 g) were dispersed in 20 mL deionized water and then heated at 65 °C for 16 h under stirring. The mixed solution was then dried at 100 °C for 20 h, and the remaining solid product was annealed at 900 °C for 1 h in argon. Finally, the sample was washed with 2 M hydrochloric acid and then with deionized water until the pH value was 7. The activated carbon was obtained after the subsequent filtration and drying process.

### 2.2. Materials Characterization

The surface morphologies of the as-prepared samples were obtained using scanning electron microscopy (SEM, Hitachi S–5200). The crystal structure of the samples was studied by X-ray diffraction (XRD, D/max 2200/PC) using Cu Kα radiation with λ = 1.5046 Å. The specific surface area and pore features were investigated by means of nitrogen adsorption/desorption isotherms with an Autosorb–iQ system. The surface chemical state of the samples was analyzed by employing X-ray photoelectron spectroscopy (XPS, ESCALAB 250Xi). The functional groups of samples were obtained using Fourier transform infrared (FTIR, Nicolet iS50). Thermogravimetric analysis (TGA) (TG 8121 Rigaku Corp) was used at a heating rate of 10 °C min^−1^ in nitrogen flow.

### 2.3. Adsorption Experiments

Adsorption experiments were carried out using activated carbon prepared by waste coffee as the adsorbent. RB was used as the adsorbate to prepare a stock solution of concentration of 20.9 mmol L^−1^ (10 g L^−1^). This solution was used to prepare the required testing samples with the concentration of 208.7 to 626 μmol L^−1^ (100 to 300 mg L^−1^). RB adsorption experiments were conducted in 500 mL glass flasks. A 200 mL RB aqueous solution containing 0.2 g activated carbon was poured into a glass flask. After that, the glass flask was covered and placed in a magnetic stirrer at 100 rpm and 50 °C. The concentration of the mixture solution was then determined at defined intervals of time using an ultraviolet visible spectrophotometer (LAMBDA 950). The maximum absorbance of RB was shown at a wavelength of 553 nm. A calibration curve was carried out for a series of RB solutions with specified concentrations, and the residual RB concentration of solution was obtained using the calibration curve. Amounts of 1 M HCl and 0.1 M KOH were used to adjust the pH of the RB solution. 

The series of values were fitted using Langmuir and Freundlich isotherm models, which are shown with the equations [23,24,27] below:

(1) $$\mathrm{Langmuir}:\;\frac{C_{e}}{Q_{e}}=\frac{1}{Q_{m}K_{L}}+\frac{1}{Q_{m}}C_{e}$$

(2) $$\mathrm{Freundlich}:\;\log Q_{e}=\log K_{F}+\frac{1}{n}\log C_{e}$$

where Q_m_ (mg g^−1^) refers to the maximum adsorption capacity of RhB; KL (L mg^−1^) represents the Langmuir adsorption constant that is associated with the adsorption affinity; and KF ((mg g^−1^) (L g^−1^)1/n) and n correspond to the Freundlich constants.

Pseudo-first-order and pseudo-second-order kinetic models are employed to investigate the isotherm kinetic, as revealed in the equations [27] below:

(3) $$\mathrm{Pseudo}-\mathrm{first}\;\mathrm{order}:\;\ln\left(Q_{e}-Q_{t}\right)=\ln Q_{e}-{\;K}_{1}t$$

(4) $$\mathrm{Pseudo}-\mathrm{second}\;\mathrm{order}:\;\frac{t}{Q_{t}}=\frac{1}{K_{2}Q_{e}^{2}}+\frac{1}{Q_{e}}t$$

where Q_e_ (mg g^−1^) and Q_t_ (mg g^−1^) correspond to the amount of RB adsorbed in activated carbon at equilibrium and at time t, respectively; t (min) refers to the contact time; and K_1_ and K_2_ represent the rate constants of pseudo-first order and pseudo-second order, respectively.

## 3. Results and Discussion

### 3.1. Physical Properties of The Activated Carbon

**Figure 1:**
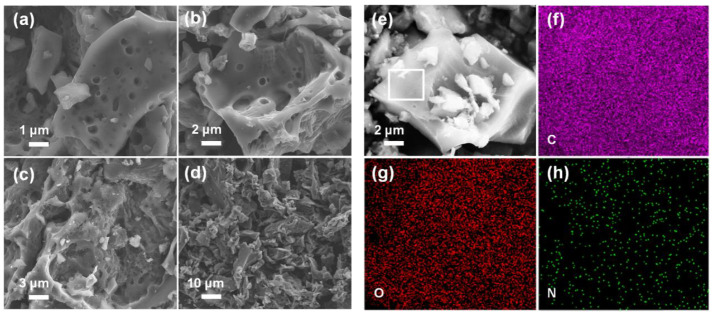
(**a**–**d**) SEM images of the activated carbon with different scales. (**e**–**h**) SEM images and EDS elemental maps of the activated carbon.

SEM and EDS measurements were used to examine the morphology, microstructure and element composition of the as-prepared activated carbon. From the SEM images (Figure 1a–d) of the different scales, it can be seen that the sample exhibits a slightly wrinkled and rough surface. The special structure can help to improve the surface area of activated carbon. Figure 1e–h show that the as-prepared activated carbon contains three elements of carbon, oxygen and nitrogen, indicating that the functional groups containing oxygen and nitrogen exist on the surface of the activated carbon. 

**Figure 2:**
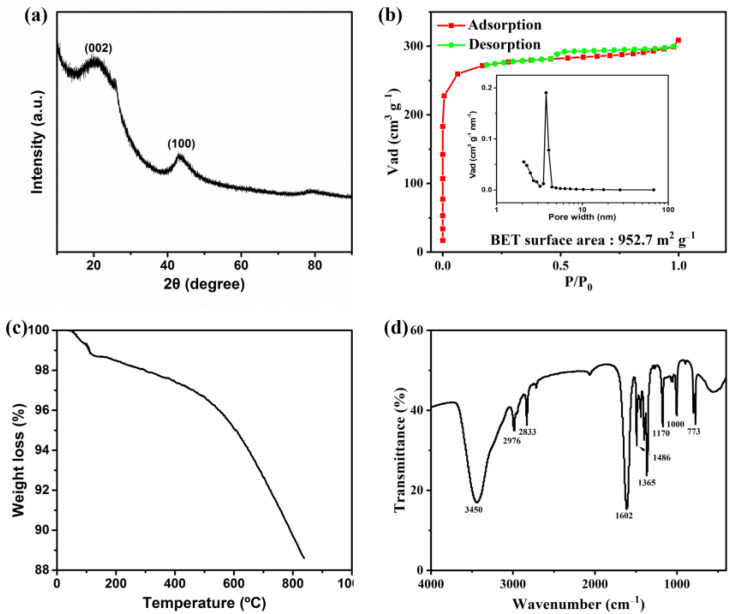
(**a**) XRD pattern of the activated carbon. (**b**) Nitrogen adsorption–desorption curves and pore size distribution of the activated carbon. (**c**) TGA and (**d**) FTIR spectra of the activated carbon.

Figure 2a shows the XRD pattern of the activated carbon. It is obvious that the XRD pattern exhibits two broad peaks of (002) at 22.3° and (100) at 42.9°, which are assigned to the layered carbon and ordered hexagonal carbon, respectively [28]. The nitrogen adsorption–desorption isotherms and the corresponding pore size distribution of the sample were calculated by the Barrett–Joyner–Halenda (BJH) method, and the results are shown in Figure 2b. It indicates a type-I isotherm, which is a typical characteristic of microporous material conforming to the pore size distribution, and the surface area of the sample is about 952.7 m^2^ g^−1^. This unique porous feature contributes to the improvement of the adsorption properties of the activated carbon. 

Figure 2c shows the thermogravimetric (TG) curves of the activated carbon prepared from waste coffee in a nitrogen flow. The thermogravimetric curves of the activated carbon consist of two main stages of dehydration and pyrolysis. The reduction in the weight of activated carbon in the range of 50–100 °C is due to the evaporation of adsorbed water. In the range of 500–900 °C, a large weight loss occurred, which should come from the decomposition of the surface functional groups of the activated carbon [29]. Figure 2d shows the FTIR spectrum of activated carbon. The broad peak at 3450 cm^−1^ is attributed to the stretching O–H bond in hydroxyl groups or NH stretch, while the peak located at (1640 to 1560) cm^−1^ shows the asymmetric stretching of the carboxylate (–COO–) group [10]. The peaks at 2976 and 2833 cm^−1^ may be attributed to the presence of C–H stretch, and the peak at 1480 cm^−1^ indicates the C=C bonds. The FTIR spectrum of the activated carbon shows major peaks at 1365, 1170, 1000 and 773 cm^−1^, which correspond to the presence of C–C bonds, C–O stretching vibration, C–N stretching and C–H bonds [30,31,32]. 

**Figure 3:**
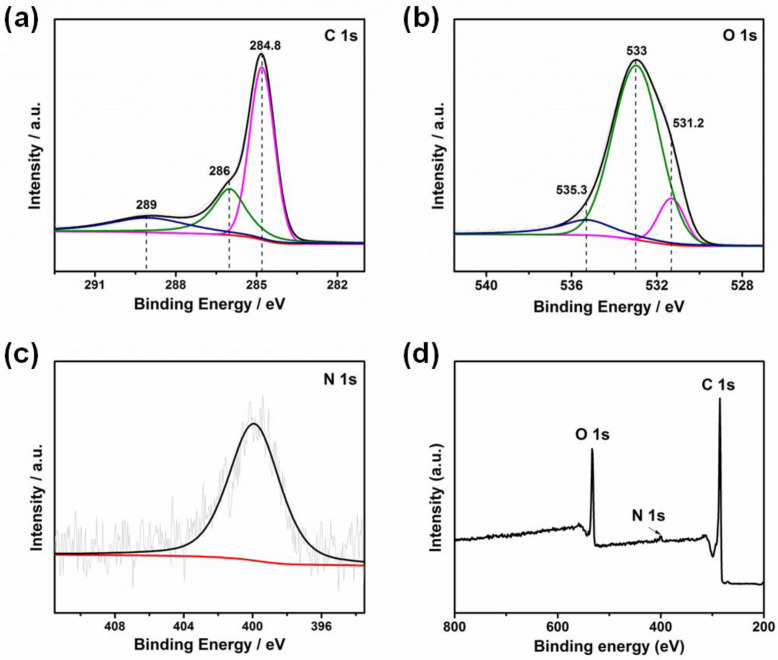
(**a**) C 1s, (**b**) O 1s, (**c**) N 1s and (**d**) survey XPS spectra of activated carbon.

To further confirm the surface functional groups on the as-prepared activated carbon, XPS analysis was investigated. Figure 3 shows the C 1s (a), O 1s (b), N 1s (c) and XPS survey spectra (d) of the activated carbon. The C 1s spectrum of the activated carbon usually consists of three types of carbon bonds: C–C or C=C (284.8 eV), C–O (286 eV) and O–C=O (289 eV) [29,30,31,32,33,34]. The O 1 s peaks could be fitted to three curves: C=O at 531.2 eV, O–H or C–O–C at 533 eV and O–C=O at 535.3 eV [32]. The contents of carbon, oxygen and nitrogen on the surface of the carbon material were also obtained, which were 79.7, 18.2 and 2.1%, respectively. In addition, the content of carboxyl on the surface of the activated carbon was 19.4%. The carboxyl and hydroxyl groups could dissociate and then be negatively charged [35]. They could then be active sites in the adsorption process. 

### 3.2. Adsorption Properties Investigation 

#### 3.2.1. The Effect of Initial RB Concentration

**Figure 4:**
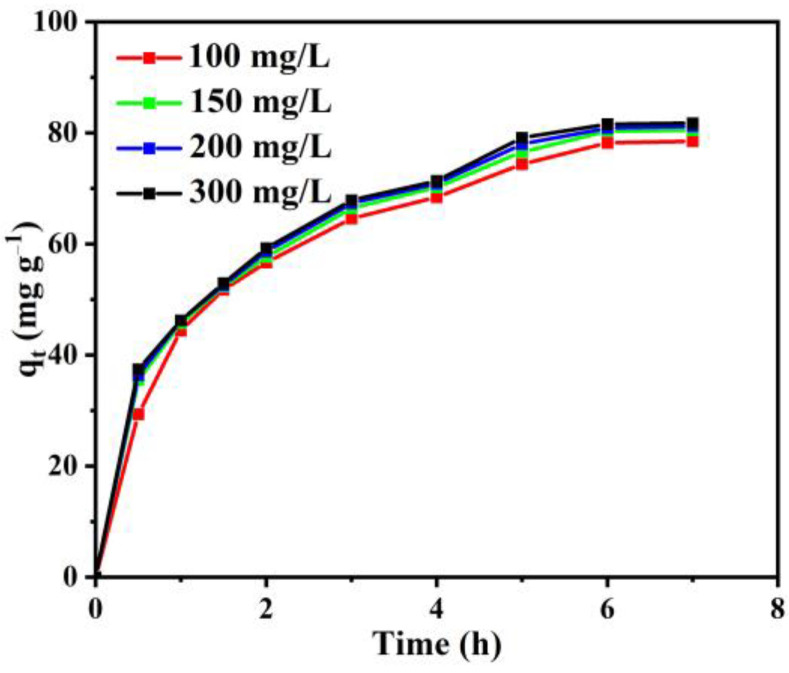
Effect of initial RB concentration on RB removal efficiency at pH = 5 and T = 50 °C.

Figure 4 shows the effect of initial RB concentration on absorption efficiency. These results revealed that in the first 3 h, the amount of RB adsorbed increased rapidly, and the adsorption on activated carbon no longer changed significantly after 6 h. Therefore, the corresponding time could be considered as the equilibrium time. The absorb ability of the activated carbon to RB is increased by only 3.3 mg g^−1^, with the initial concentration of RB increasing from 100 to 300 mg L^−1^. This result indicated that a further increase in the concentration of RB does not affect the uptake capacity significantly due to the saturation of the adsorption sites [36]. 

Adsorption isotherm models of Langmuir and Freundlich models were applied to evaluate the performance of the as-prepared activated carbon on the removal of RB from aqueous solution, and the corresponding parameters are shown in Table 1 (Equations (1) and (2)). For all of the isotherm models, the Langmuir model presents the results better than the Freunflich model with a higher R^2^, suggesting a monolayer process for the activated carbon toward the adsorption of RB [37].

#### 3.2.2. The Effect of Temperature

**Figure 5:**
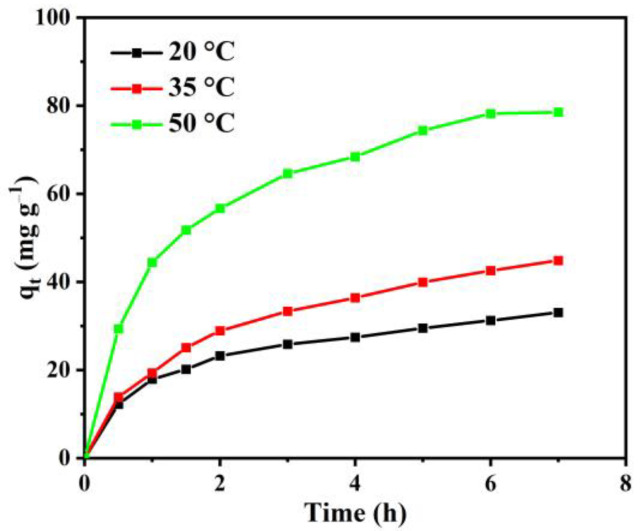
The effect of temperature on uptake capacity of activated carbon at an initial RB concentration of 100 mg L^−1^ and pH = 5.

In order to research the effect of solution temperature on the uptake capacity of the activated carbon, a bath experiment with different temperatures was carried out. The results are shown in Figure 5. Obviously, the amount of RB adsorbed per unit mass of the activated carbon increased with the temperature from 20 to 50 °C, indicating that the uptake capacity of the activated carbon depends on temperature significantly. 

As analyzed above, the obtained activated carbon displays a microporous feature, and the unique pore size distribution can lead to an increase in the adsorption capacity with increases in temperature. The molecular size of RB is nearly 2 nm. Therefore, after the pore has been adsorbed by the RB molecule at the entrance, it will impede the subsequent adsorption of RB molecules. The diffusion rate of RB molecules into the pores will be enhanced with increasing temperatures due to the endothermic diffusion process [38]. Therefore, the uptake capacity of the activated carbon improved to some extent as the temperature increased.

#### 3.2.3. The Effect of Solution PH

**Figure 6:**
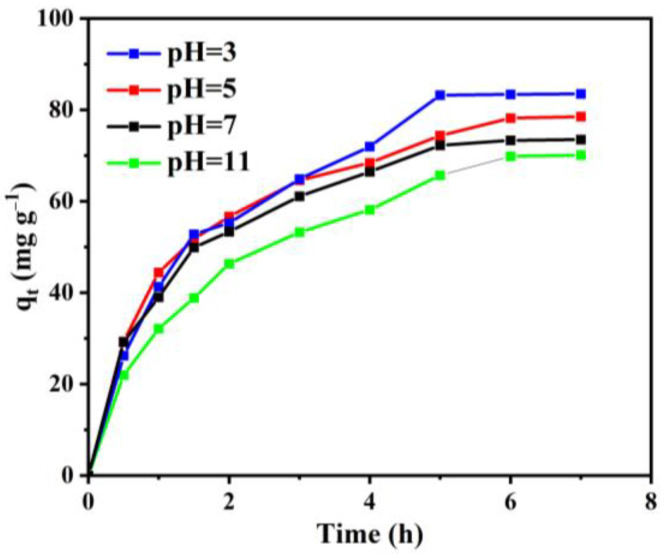
Effect of solution pH on uptake capacity of activated carbon at initial RB concentration of 100 mg L^−1^ and T=50°C.

To investigate the effect of solution pH on the uptake capacity of the activated carbon, a series of adsorption experiments with a different pH (from 3 to 11) were carried out, where the initial RB concentration was 100 mg L^−1^ and the temperature was 50 °C. The obtained results are shown in Figure 6, and demonstrate that the uptake capacity of the activated carbon was changed as the pH increased from 3 to 11. The maximum value of the adsorption capacity occurred at pH 7, which was 83.4 mg g^−1^. Compared with some reported materials (e.g., ZIF–8, ZIF–67, ZIF–8@ZIF–67 [39]; polyamide grafted carbon microspheres [40]), the adsorption capacity of the activated carbon delivers a relatively desirable level. 

To investigate the mechanism of the adsorption process, the kinetic data and the related kinetic parameters were simulated by employing both the typical pseudo-first-order and pseudo-second-order kinetic models (Table 2) (Equations (3) and (4)). The parameters obtained from the adsorption of RB onto activated carbon based on the pseudo-second-order kinetic model match our experimental results well, with the highest R^2^.

From the XPS results, there are certain surface functional groups on the as-prepared activated carbon, including the carboxyl and hydroxyl groups. These surface functional groups could dissociate and then be negatively charged [35], thus becoming active sites. 

The interaction between the negatively charged functional groups in the activated carbon and the positively charged functional groups in RB enhances the adsorption capacity of the activated carbon. Therefore, the adsorption capacity of the adsorbent is significantly affected by the solution pH. In the acidic solution, the amount of H^+^ will compete with RB molecules for the adsorption sites of the activated carbon [41]. Therefore, the uptake capacity of the activated carbon is decreased in the acidic solution. Moreover, the RB molecules will be transformed from cationic to zwitterionic by deprotonation of the carboxyl group if the value of pH is greater than 3.7 [13]. For this reason, the electrostatic interaction between the negatively charged activated carbon and the zwitterionic RB will be weakened, and then the uptake capacity of the activated carbon is decreased as the value of pH is increased. Due to the above two reasons, the adsorption capacity of the activated carbon changed with pH, as shown in Figure 6.

## 4. Conclusions

In summary, we synthesized a low-cost activated carbon via physiochemical activation using waste coffee as a potential raw material.

The obtained activated carbon possesses a large surface area of about 952.7 m^2^ g^−1^ and can be used as an adsorbent to remove the RB from aqueous solution successfully. The maximum uptake capacity was achieved under the following appropriate conditions: an initial concentration of 100 mg L^−1^, a temperature of 50°C and a pH value of 7. The enhanced adsorption properties of the activated carbon can be attributed to its unique porous carbon structure, especially for the negatively charged surface functional groups, which can promote strong electrostatic attraction with positively charged functional groups in RB. This study demonstrates that waste-coffee-derived activated carbon could be used as an alternative low-cost adsorbent to commercial activated carbon for the removal of various toxic dyes from aqueous solution. 

## Figures and Tables

**Table 1:** Adsorption isotherm constants for RB adsorption onto the activated carbon (Equations (1) and (2)).

Sample	Isotherm Models					
	Langmuir			Freundlich		
	Q_m_ (mg g^−1^)	K_L_ (L mg^−1^)	R^2^	K_F_ ((mg g^−1^) (L/mg)^1/n^)	1 /n	R^2^
Activated Carbon	83.3	0.86	0.999	74.3	0.018	0.975

**Table 2:** Parameters of kinetic models for the adsorption of RB onto the activated carbon (Equations (3) and (4)).

Kinetic Models	Pseudo-First Order				Pseudo-Second Order		
Parameters	Q_e,exp_ (mg g^−1^)	Q_e,cal_ (mg g^−1^)	K_1_ (min^−1^)	R^2^	Q_e,cal_ (mg g^−1^)	K_2_ (mg min^−1^)	R^2^
50°C	80.5	60.3	0.0083	0.974	88.0	1.7 × 10^−4^	0.997
35°C	44.9	36.6	0.0065	0.998	51.0	2.2 × 10^−4^	0.994
20°C	33.1	22.2	0.0061	0.984	34.5	4.9 × 10^−4^	0.998
pH = 5	70.1	64.1	0.0081	0.956	81.2	1.3 × 10^−4^	0.990
pH = 7	83.5	66.7	0.0073	0.984	94.6	9.8 × 10^−5^	0.991
pH = 11	73.5	54.6	0.0086	0.993	82.6	1.7 × 10^−4^	0.994

## Data Availability

Not applicable.
